# Supplementary material for: External validation of the priapism impact profile in a Jamaican cohort of patients with sickle cell disease
Source: PLoS One. 2021 Oct 15;16(10):e0258560. doi: 10.1371/journal.pone.0258560 (PMC8519460; doi:10.1371/journal.pone.0258560)
Supplement: S1 File — (DOCX) [file pone.0258560.s001.docx]

**Supporting Information**

**S1 File- Priapism Impact Profile Questionnaire**

**Priapism Impact Profile (PIP)**

(Version 2.0 Drafted 10/14/13)

**Instructions**

*B Below are some questions we would like you to answer about your problems with priapism and*

*how you have been feeling during the past 2 weeks. If you are confused or have any problems*

*i with the questions please ask for help from the study staff, and someone will be happy to assist*

*y you. Please circle the number that best describes your personal experience, and please do not skskip any items.*

***DDuring the past 2 weeks:***

| **1.** Worry about my overall health has been: | **None** | **Minimal** | **Slight** | **Moderate** | **Substantial** | **Extreme** | **Very Extreme** |
| --- | --- | --- | --- | --- | --- | --- | --- |
|  | **1** | **2** | **3** | **4** | **5** | **6** | **7** |
| **2.** My distress about my priapism has been: | **None** | **Minimal** | **Slight** | **Moderate** | **Substantial** | **Extreme** | **Very Extreme** |
|  | **1** | **2** | **3** | **4** | **5** | **6** | **7** |
| **3.** The effect of priapism on my daily activities has been: | **None** | **Minimal** | **Slight** | **Moderate** | **Substantial** | **Extreme** | **Very Extreme** |
|  | **1** | **2** | **3** | **4** | **5** | **6** | **7** |
| **4.** The negative effect of priapism on my feelings have been: | **None** | **Minimal** | **Slight** | **Moderate** | **Substantial** | **Extreme** | **Very Extreme** |
|  | **1** | **2** | **3** | **4** | **5** | **6** | **7** |
| **5.** The effect of priapism on my sexual satisfaction has been: | **None** | **Minimal** | **Slight** | **Moderate** | **Substantial** | **Extreme** | **Very Extreme** |
|  | **1** | **2** | **3** | **4** | **5** | **6** | **7** |
| **6.** The effect of priapism on my relationship with my partner has been: | **None** | **Minimal** | **Slight** | **Moderate** | **Substantial** | **Extreme** | **Very Extreme** |
|  | **1** | **2** | **3** | **4** | **5** | **6** | **7** |
| **7.** The effect of priapism on my sexual confidence has been: | **None** | **Minimal** | **Slight** | **Moderate** | **Substantial** | **Extreme** | **Very Extreme** |
|  | **1** | **2** | **3** | **4** | **5** | **6** | **7** |
| **8.** Having trouble getting an erection has been: | **None** | **Minimal** | **Slight** | **Moderate** | **Substantial** | **Extreme** | **Very Extreme** |
|  | **1** | **2** | **3** | **4** | **5** | **6** | **7** |
| **9.** Problems with my sexual desire have been: | **None** | **Minimal** | **Slight** | **Moderate** | **Substantial** | **Extreme** | **Very Extreme** |
|  | **1** | **2** | **3** | **4** | **5** | **6** | **7** |
| **10.** Physical discomfort caused by my priapism has been: | **None** | **Minimal** | **Slight** | **Moderate** | **Substantial** | **Extreme** | **Very Extreme** |
|  | **1** | **2** | **3** | **4** | **5** | **6** | **7** |
| **11.** The level of pain in my penis caused by my priapism has been: | **None** | **Minimal** | **Slight** | **Moderate** | **Substantial** | **Extreme** | **Very Extreme** |
|  | **1** | **2** | **3** | **4** | **5** | **6** | **7** |
| **12.** The abnormal shape of my penis caused by priapism has been: | **None** | **Minimal** | **Slight** | **Moderate** | **Substantial** | **Extreme** | **Very Extreme** |
|  | **1** | **2** | **3** | **4** | **5** | **6** | **7** |

*Plea*

*se* ***r Read the following list*** *of questions and circle the response that best rates your opinion regarding each item item’s importance and clarity.*

| **1.** Worry about my overall health has been: | To me this question was of: **High Importance \| Medium Importance \| Low Importance** |
| --- | --- |
|  | To me, this question was of: **High Clarity \| Medium Clarity \| Low Clarity** |
| **2.** My distress about my priapism has been: | To me this question was of: **High Importance \| Medium Importance \| Low Importance** |
|  | To me, this question was of: **High Clarity \| Medium Clarity \| Low Clarity** |
| **3.** The effect of priapism on my daily activities has been: | To me this question was of: **High Importance \| Medium Importance \| Low Importance** |
|  | To me, this question was of: **High Clarity \| Medium Clarity \| Low Clarity** |
| **4.** The negative effect of priapism on my feelings have been: | To me this question was of: **High Importance \| Medium Importance \| Low Importance** |
|  | To me, this question was of: **High Clarity \| Medium Clarity \| Low Clarity** |
| **5.** The effect of priapism on my sexual satisfaction has been: | To me this question was of: **High Importance \| Medium Importance \| Low Importance** |
|  | To me, this question was of: **High Clarity \| Medium Clarity \| Low Clarity** |
| **6.** The effect of priapism on my relationship with my partner has been: | To me this question was of: **High Importance \| Medium Importance \| Low Importance** |
|  | To me, this question was of: **High Clarity \| Medium Clarity \| Low Clarity** |
| **7.** The effect of priapism on my sexual confidence has been: | To me this question was of: **High Importance \| Medium Importance \| Low Importance** |
|  | To me, this question was of: **High Clarity \| Medium Clarity \| Low Clarity** |
| **8.** Having trouble getting an erection has been: | To me this question was of: **High Importance \| Medium Importance \| Low Importance** |
|  | To me, this question was of: **High Clarity \| Medium Clarity \| Low Clarity** |
| **9.** Problems with my sexual desire have been: | To me this question was of: **High Importance \| Medium Importance \| Low Importance** |
|  | To me, this question was of: **High Clarity \| Medium Clarity \| Low Clarity** |
| **10.** Physical discomfort caused by my priapism has been: | To me this question was of: **High Importance \| Medium Importance \| Low Importance** |
|  | To me, this question was of: **High Clarity \| Medium Clarity \| Low Clarity** |
| **11.** The level of pain in my penis caused by my priapism has been: | To me this question was of: **High Importance \| Medium Importance \| Low Importance** |
|  | To me, this question was of: **High Clarity \| Medium Clarity \| Low Clarity** |
| **12.** The abnormal shape of my penis caused by priapism has been: | To me this question was of: **High Importance \| Medium Importance \| Low Importance** |
|  | To me, this question was of: **High Clarity \| Medium Clarity \| Low Clarity** |

| **Scoring** | | | |
| --- | --- | --- | --- |
| **Domain** | **Quality of Life** | **Sexual Function** | **Physical Impact** |
| **Questions** | 1. ___________  2. ___________  3. ___________  4. ___________ | 5. ____________  6. ____________  7. ____________  8. ____________  9. ____________ | 10. ____________  11. ____________  12. ____________ |
| **Total Score** | ______________  (4-28) | ______________  (5-35) | ______________  (3-21) |

Total PIP Score ( ∑ Items 1-12) = _________

| **Rating** | | |
| --- | --- | --- |
| **Number of Questions with** | **Importance** | **Clarity** |
| **High** | ___________ | ____________ |
| **Medium** | ___________ | ____________ |
| **Low** | ___________ | ____________ |

**S1 Table -Paired T-Test comparing scores on questions 1-12 for Retest versus original**

|  | **Paired T-Test^a^ comparing scores on Q1 - Q12 for RETEST vs. Original** | | | | | |  |
| --- | --- | --- | --- | --- | --- | --- | --- |
| **Questions** | **# of paired subjects** | **Mean^b^ difference** | **%95 CI** | **Min** | **Max** | **p-value** |  |
| Q1 | 20 | 0.10 | (-0.62, 0.82) | -2 | 3 | 0.78 |  |
| Q2 | 20 | -0.40 | (-1.36, 0.56) | -6 | 3 | 0.40 |  |
| Q3 | 20 | -0.40 | (-1.10, 0.30) | -3 | 3 | 0.25 |  |
| Q4 | 20 | 0.10 | (-0.40, 0.60) | -2 | 2 | 0.68 |  |
| Q5 | 20 | -0.25 | (-0.83, 0.33) | -3 | 2 | 0.38 |  |
| Q6 | 20 | -0.25 | (-0.77, 0.27) | -2 | 1 | 0.33 |  |
| Q7 | 20 | -0.30 | (-1.10, 0.50) | -4 | 3 | 0.44 |  |
| Q8 | 20 | 0.05 | (-0.30, 0.40) | -1 | 2 | 0.77 |  |
| Q9 | 19 | -0.53 | (-1.35, 0.29) | -5 | 2 | 0.20 |  |
| Q10 | 20 | -0.30 | (-1.47, 0.87) | -4 | 5 | 0.60 |  |
| Q11 | 19 | -0.58 | (-1.58, 0.42) | -5 | 3 | 0.24 |  |
| Q12 | 19 | -0.42 | (-1.38, 0.54) | -5 | 3 | 0.37 |  |
| Notes: ^a^ Because each subject had responses on the same questionnaires at an initial time point and a later RETEST time point, Paired T-Test was used to detect the difference between the means | | | | | | | |
| ^b^ Mean difference reflects RETEST score – Original score | | | | | | | |
|  | | | | | | | |

**S2 Table- Paired T-Test comparing scores on PIP, QoL, Sexual function for Retest versus original**

|  | **Paired T-Test^a^ comparing scores on PIP, QoL, Sexual function for RETEST vs. Original** | | | | | |  |
| --- | --- | --- | --- | --- | --- | --- | --- |
| **Measures derived from Q1-Q12** | **# of paired subjects** | **Mean difference^b^** | **%95 CI** | **Min** | **Max** | **p-value** |  |
| Total PIP | 20 | -3.25 | (-8.29, 1.79) | -21 | 22 | 0.19 |  |
| Quality of life(QoL) | 20 | -0.60 | (-2.51, 1.31) | -8 | 11 | 0.52 |  |
| Sexual function | 20 | -1.45 | (-3.66, 0.76) | -9 | 7 | 0.19 |  |
| Physical impact | 20 | -1.20 | (-3.39, 0.99) | -9 | 9 | 0.27 |  |
| Notes: ^a^ Because each subject had responses on the same questions at an initial time point and a later RETEST time point, Paired T-Test was used to detect the difference between the means | | | | | | | |

**S3 Table- McNemar Chi Square test comparing the proportion of responses to question importance on Retest versus original**

|  | | **Responses to Question importance at Original test** | | **Responses to Question importance at RE-test** | |  |  |
| --- | --- | --- | --- | --- | --- | --- | --- |
| **Question Importance** | **# of paired subjects** | **L n (%)** | **H or M n (%)** | **L n (%)** | **H or M n (%)** | **p-value^a^** |  |
| Q1 | 20 | 2 (10%) | 18 (90%) | 2 (10%) | 18 (90%) | 1.00 |  |
| Q2 | 19 | 2 (11%) | 17 (89%) | 4 (21%) | 15 (79%) | 0.31 |  |
| Q3 | 20 | 2 (10%) | 18 (90%) | 7 (35%) | 13 (65%) | 0.05 |  |
| Q4 | 20 | 3 (15%) | 17 (85%) | 5 (25%) | 15 (75%) | 0.47 |  |
| Q5 | 20 | 6 (30%) | 14 (70%) | 4 (20%) | 16 (80%) | 0.47 |  |
| Q6 | 20 | 6 (30%) | 14 (70%) | 5 (25%) | 15 (75%) | 0.73 |  |
| Q7 | 20 | 6 (30%) | 14 (70%) | 7 (35%) | 13 (65%) | 0.70 |  |
| Q8 | 20 | 6 (30%) | 14 (70%) | 6 (30%) | 14 (70%) | 1.00 |  |
| Q9 | 19 | 6 (32%) | 13 (68%) | 3 (16%) | 16 (84%) | 0.31 |  |
| Q10 | 19 | 2 (11%) | 17 (89%) | 2 (11%) | 17 (89%) | 1.00 |  |
| Q11 | 20 |  | 20 (100%) | 1 (5%) | 19 (95%) | NA |  |
| Q12 | 16 | 5 (31%) | 11 (69%) | 5 (31%) | 11 (69%) | 1.00 |  |
| Notes: ^a^ p-values were based on McNemar Chi-Square test comparing the proportion of responses to question importance on RETEST vs. Original | | | | | | | |

**S4 Table- McNemar Chi Square test comparing the proportion of responses to question clarity on Retest versus original**

|  | | **Responses to Question clarity at Original test** | | **Responses to Question clarity at RE-test** | |  |  |
| --- | --- | --- | --- | --- | --- | --- | --- |
| **Question Clarity** | **# of paired subjects** | **L n (%)** | **H or M n (%)** | **L n (%)** | **H or M n (%)** | **p-value^a^** |  |
| Q1 | 20 |  | 20 (100%) | 1 (5%) | 19 (95%) | NA |  |
| Q2 | 19 | 1 (5%) | 18 (95%) | 1 (5%) | 18 (95%) | 1.00 |  |
| Q3 | 20 | 1 (5%) | 19 (95%) | 3 (15%) | 17 (85%) | 0.31 |  |
| Q4 | 20 | 4 (20%) | 16 (80%) | 2 (10%) | 18 (90%) | 0.31 |  |
| Q5 | 20 | 3 (15%) | 17 (85%) | 1 (5%) | 19 (95%) | 0.15 |  |
| Q6 | 20 | 2 (10%) | 18 (90%) | 1 (5%) | 19 (95%) | 0.31 |  |
| Q7 | 20 | 2 (10%) | 18 (90%) | 2 (10%) | 18 (90%) | 1.00 |  |
| Q8 | 20 | 2 (10%) | 18 (90%) | 1 (5%) | 19 (95%) | 0.31 |  |
| Q9 | 19 | 3 (16%) | 16 (84%) | 1 (5%) | 18 (95%) | 0.15 |  |
| Q10 | 19 | 1 (5%) | 18 (95%) | 1 (5%) | 18 (95%) | 1.00 |  |
| Q11 | 20 |  | 20 (100%) |  | 20 (100%) | NA |  |
| Q12 | 16 | 4 (25%) | 12 (75%) | 3 (19%) | 13 (81%) | 0.31 |  |
| Notes: ^a^ p-values were based on McNemar Chi-Square test comparing the proportion of responses to question clarity on RETEST vs. Original | | | | | | | |

**S5 Table- Weighted average of the proportion of responses H or M to question importance on Q1 - Q12 for Retest versus original**

|  | **Original test** | | | | **RE-test** | | | |
| --- | --- | --- | --- | --- | --- | --- | --- | --- |
| **Question importance** | **# of samples** | **# of H or M responses** | **% of H or M responses** | **Weighted average of % of H or M responses on Q1-Q12** | **# of samples** | **# of H or M responses** | **% of H or M responses** | **Weighted average of % of H or M responses on Q1-Q12** |
| Q1 | 20 | 18 | 90.0% | 79.7% | 20 | 18 | 90.0% | 77.5% |
| Q2 | 20 | 18 | 90.0% |  | 19 | 15 | 78.9% |  |
| Q3 | 20 | 18 | 90.0% |  | 20 | 13 | 65.0% |  |
| Q4 | 20 | 17 | 85.0% |  | 20 | 15 | 75.0% |  |
| Q5 | 20 | 14 | 70.0% |  | 20 | 16 | 80.0% |  |
| Q6 | 20 | 14 | 70.0% |  | 20 | 15 | 75.0% |  |
| Q7 | 20 | 14 | 70.0% |  | 20 | 13 | 65.0% |  |
| Q8 | 20 | 14 | 70.0% |  | 20 | 14 | 70.0% |  |
| Q9 | 20 | 14 | 70.0% |  | 19 | 16 | 84.2% |  |
| Q10 | 19 | 17 | 89.5% |  | 20 | 17 | 85.0% |  |
| Q11 | 20 | 20 | 100% |  | 20 | 19 | 95.0% |  |
| Q12 | 18 | 11 | 61.1% |  | 18 | 12 | 66.7% |  |

**S6 Table- Weighted average of the proportion of responses H or M to question clarity on Q1 - Q12 for Retest versus Original**

|  | **Original test** | | | | **RE-test** | | | |
| --- | --- | --- | --- | --- | --- | --- | --- | --- |
| **Question clarity** | **# of samples** | **# of H or M responses** | **% of H or M responses** | **Weighted average of % of H or M responses on Q1-Q12** | **# of samples** | **# of H or M responses** | **% of H or M responses** | **Weighted average of % of H or M responses on Q1-Q12** |
| Q1 | 20 | 20 | 100% | 89.9% | 20 | 19 | 95.0% | 92.8% |
| Q2 | 20 | 19 | 95.0% |  | 19 | 18 | 94.7% |  |
| Q3 | 20 | 19 | 95.0% |  | 20 | 17 | 85.0% |  |
| Q4 | 20 | 16 | 80.0% |  | 20 | 18 | 90.0% |  |
| Q5 | 20 | 17 | 85.0% |  | 20 | 19 | 95.0% |  |
| Q6 | 20 | 18 | 90.0% |  | 20 | 19 | 95.0% |  |
| Q7 | 20 | 18 | 90.0% |  | 20 | 18 | 90.0% |  |
| Q8 | 20 | 18 | 90.0% |  | 20 | 19 | 95.0% |  |
| Q9 | 20 | 17 | 85.0% |  | 19 | 18 | 94.7% |  |
| Q10 | 19 | 18 | 94.7% |  | 20 | 19 | 95.0% |  |
| Q11 | 20 | 20 | 100% |  | 20 | 20 | 100% |  |
| Q12 | 18 | 13 | 72.2% |  | 18 | 15 | 83.3% |  |
